# Validity of the 6-minute walk test and step test for evaluation of cardio respiratory fitness in patients with type 2 diabetes mellitus

**DOI:** 10.20463/jenb.2018.0008

**Published:** 2018-03-31

**Authors:** Min Chul Lee

**Affiliations:** 1. Department of Sports Medicine , CHA University of College of Health Science, Pocheon Republic of Korea

**Keywords:** type 2 diabetes mellitus, 6-minute walk test, step test, cardiorespiratory fitness

## Abstract

**[Purpose]:**

The purpose of this study was to evaluate the reliability, reproducibility, and validity of the 6-minute walk test and step test as substitutes for ergometer exercise in patients with type 2 diabetes mellitus.

**[Methods]:**

The study included 54 women aged 50–70 years who had type 2 diabetes mellitus. All the patients performed a cycle ergometer-graded exercise test for cardiorespiratory fitness (${\dot{V}O}_{2max}$), followed by 6-minute walk and step tests.

**[Results]:**

The mean distance covered during the 6-minute walk test was 538 ± 54 m, and the mean recovery heart rate after the step test was 93 ± 11 beats/min. A significant correlation was found between the distances covered during the cardiorespiratory fitness test (${\dot{V}O}_{2max}$) and those covered the 6-minute walk test (*r* = 0.542, *p* < 0.01) and step test (*r* = −0.490, *p* < 0.01) in the patients with type 2 diabetes mellitus. Regression equations for the prediction of ${\dot{V}O}_{2max}$ were constructed from the distance covered, heart rate, age, weight, height, fasting blood glucose level, 2-hour postprandial glucose level, and glycated hemoglobin level during the 6-minute walk test and step test.

**[Conclusion]:**

The 6-minute walk and step tests are simple to perform and reliable for the evaluation of cardiorespiratory fitness in patients with type 2 diabetes mellitus.

## INTRODUCTION

Recently, the number of patients with diabetes mellitus has been increasing worldwide owing to economic and social developments, improvements in living standards, and increase in the prevalence of obesity and stress. Particularly, the prevalence of diabetes mellitus, which had been uncommon in Korea in the past, has been rapidly increasing with social and economic developments, diabetic patients, who were uncommon in Korea in the past, are rapidly increasing in number. Its prevalence, estimated to be <1% in the 1970s, increased to approximately 3% in the 1980s, and estimates for the 2000s vary from 7% to >12% in advanced countries^1^. As the prevalence of this disease increased, the mortality rate per 100,000 populations increased from 4.3 to 17.2 deaths in 1994, 21.8 in 1999, and 24.3 in 2017, showing a linear increase^2^.

Diabetes mellitus is an aggregation of all metabolic diseases with hyperglycemia due to increased glucose level in the blood. It is a chronic metabolic disease of carbohydrates, proteins, and fats characterized by hyperglycemia due to insufficient secretion and impaired utilization of insulin. Although not a direct cause of death, diabetes mellitus yields serious damages by causing blindness, kidney failure, non-traumatic limb amputation, and complications such as coronary artery disease and stroke^3^. Therefore, as a chronic disease whose symptoms can be controlled but cannot be cured, the goal of diabetes mellitus management, which is a lifelong task, is to improve symptoms, maintain normal blood glucose levels, and prevent acute and chronic complications^4^.

In non-insulin-dependent diabetes mellitus, adequate dietary therapy and exercise alone can effectively correct metabolic abnormalities3. In particular, exercise therapy has few side effects and is a long-term approach to the fundamental treatment of all related metabolic disorders, unlike medication or diet. This therapy is an important treatment for adult diseases caused by metabolic diseases, which can be more effective when combined with medication and diet. Exercise programs and dietary regimens to control type 2 diabetic mellitus reduce the risk factors of vascular complications. The combination of these two treatments for 3 weeks decreased the fasting blood glucose levels and significantly decreased blood pressure^5^. In addition, when exercise and diet are used together, they are effective in reducing low-density lipoprotein (LDL)-cholesterol, high-density lipoprotein (HDL)-cholesterol, triglyceride (TG), and total cholesterol (TC) levels in the blood^6^. Therefore, in patients with type 2 diabetes mellitus, proper dietary and exercise therapy practices may be the key to their treatment by alleviating various possible risks.

However, many patients with diabetes mellitus have been reported to fail to practice proper diet and exercise^7,8^. This may be because they exercise without understanding their own exact fitness level. Therefore, as individuals have varying fitness levels, they should receive an exact exercise prescription according to their own fitness level. Information from physical fitness tests, including personal health and medical information, can be used by health/fitness professionals to help individuals achieve specific physical fitness goals.

In general, the maximum oxygen uptake is widely used as a key indicator of cardiopulmonary capacity assessment and to determine respiratory circulation functions such as exercise intensity when exercising^9^. There are direct and indirect methods to measure the maximum oxygen uptake. Among the direct methods, the most preferable is to obtain the maximum oxygen uptake by analyzing the exhalation gas just before the end of the exercise during imposition of an incremental load by using a treadmill or a cycle ergometer and reaching an all-out state in which the subject can no longer continue the exercise. However, the experimental equipment is expensive, requires advanced measurement technology, and is time consuming to use. In addition, strong motivation is required for the subject to reach the exhaustion state under a high intensity load, which is not suitable for individuals who do not have a habit of exercising, for the elderly, or people with illnesses.

On the contrary, the indirect method involves estimation of the oxygen uptake using data such as heart rate obtained during submaximal exercises^10^. As the indirect method is simple and economical and does not require the maximum exercise load, it can be applied to children, the elderly, and sick individuals. For these reasons, indirect methods of estimating oxygen uptake are widely used, with the step test, which uses heart rate, and the walk test, which uses the travel distance and heart rate, being the most frequently used^11^. However, many types of step tests are not height appropriate for patients with diabetes mellitus, and while the 12-minute or 2-km walk test shows high validity, it is difficult to implement for a group of individuals because it requires a large space or track, is difficult to implement in rainy weather, and is time consuming.

However, the Tecumseh step test is a low-intensity step test used in large-scale epidemiological surveys in Michigan. As the step box height is low, this test is an excellent method for predicting cardiopulmonary endurance by measuring the heart rate in response to the step and the recovery heart rate after the exercise without any discomfort to elderly or sick individuals^12^. The Tecumseh step test is performed at a rate of 24 times/min for 3 minutes by using a 20.3-cm step. As previously mentioned, it is used for large-scale epidemiological investigations because of its wide range of application from ages 10 to 69 years, and because the height of the step box is low, it does not cause strain to the elderly and patients with complications. Therefore, the step test can be conducted using a simple equipment, and is easy to perform, with no practice required. It is usually performed in a short period and is excellent for many individuals. After the exercise, the recovery heart rate decreases as cardiopulmonary endurance improves, and the test results are easy to explain to participants.

Walking has emerged as one of the most popular activities to improve fitness over the last few decades. It is popular because it is simple and can be performed by individuals of all ages and abilities owing to its low risk of injury. Walking exercise is easy to use, as it requires simple preparation and has many desirable effects such as improving aerobic capacity and reducing body fat, risks of depression and anxiety, and blood cholesterol levels^13^. In particular, the 6-minute walk test (6-MWT) is also a widely used method for evaluating cardiopulmonary performance^14^. The correlation between the 6-MWT travel distance and the maximum oxygen uptake was shown to be high (*r* = 0.73)^15^. Therefore, walking is an attractive aerobic exercise that is easily selected for exercise testing. Both above-mentioned

Both above-mentioned tests require minimum equipment, are simple, do not require practice, and are conducted in a short period. They are also an excellent method for many individuals, and the test results are easy to explain to participants. However, most previous studies have not investigated patients with diabetes mellitus aged >50 years, and no reports have described the usefulness or errors of these indirect methods when applied to such patients in Korea. Therefore, a simple, safe, and reliable method of measuring cardiovascular endurance that can be performed with minimal indoor space without requiring expensive equipment is necessary for patients with diabetes mellitus. Hence, it is meaningful to examine the validity of the maximum oxygen uptake through the 6-MWT and step test in patients with diabetes mellitus and to develop an estimation equation. Therefore, the purposes of this study was to evaluate cardiopulmonary capacity on the basis of cycle ergometer exercise test results in community-dwelling patients with type 2 diabetes mellitus aged 50–70 years, to determine the maximum oxygen uptake equation by performing the 6-MWT and Tecumseh step test, and to identify the validity and utility of the two tests for indirect measurement of maximum oxygen uptake in comparison with those of exercise load tests.

## METHODS

### Subjects and study design

The subjects of this study were patients who visited E hospital in Seoul (54 women aged 50–70 years with fasting blood glucose levels of ≥126 or <126 mg/dL but with a 2-hour postprandial blood glucose level of ≥200 mg/dL, according to the diagnostic criteria for type 2 diabetes mellitus). The other inclusion criteria were as follows: no diabetic complications, full understanding of the purpose of the study, and agreement to participate in the study^1^.

### Cardiorespiratory fitness tests

#### The cycle ergometer test

For the incremental exercise testing, the participants fasted for 12 hours. Thereafter, they were administered insulin or oral hypoglycemic agents before the exercise test the next day. Two hours after the meal, electrocardiography (ECG) was performed. The blood pressure and heart rate of the subjects during rest and the exercise test were assessed. The per-minute oxygen uptake (VO_2_), oxygen uptake (O_2_), carbon dioxide emissions (CO_2_), respiratory exchange rate, and ventilation threshold were measured on a breath-by-breath basis using JAGER (ER900, Germany) connected to a bicycle (Ergometer 900, D-72475 Bitz, Germany).

The exercise was continued by increasing the intensity by 20 W every 2 minutes until the subjects could no longer continue exercising. The bicycle pedaling speed was maintained at 50 rpm. Anaerobic threshold measurement was performed using the V-slope method on the screen showing the oxygen uptake and CO_2_ emission values on the x- and y-axes every 15 seconds by storing each measurement item record every breath during the exercise test^16^. VO_2_, work rate, heart rate, and time were measured at the ventilation threshold. The criteria for stopping exercise were based on the diagnostic criteria of the American Diabetes Association, and the examiner encouraged the subjects to continue exercising with full strength^17^.

#### The 6-minute walk test

The 6-MWT was performed in accordance with the guidelines of the American Thoracic Society^14^. The subjects were instructed to walk for 6 minutes at a given time along a 30-m line at an interval of 1.5 m in an outdoor corridor, and the distance walked was recorded in meters. The walk tests were conducted in hallways or outdoor corridors, and the patients were encouraged to continue walking as fast as possible.

#### The Tecumseh step test

The cardiopulmonary capacity of the step test was determined using the Tecumseh step test method. This low-intensity step test was performed using the method used in a large-scale epidemiological study conducted in Michigan^12^. The procedure was performed using a bench height of 20.3 cm and a rate of 24 steps per minute for 3 minutes (metronome 96/minute). After sitting and resting for 1 minute immediately after the test, the patient’s heart rate at 1:00–1:10 after the test was measured^18^.

**Table 1.** Clinical characteristics of the patients with type 2 diabetes mellitus. All data are presented as means ± standard deviations. WHR, waist hip ratio; BMI, body mass index; FBS, fasting blood glucose; PP2, 2-hour postprandial glucose.

Variable	Mean		S.D.
**Duration of DM (yrs)**	6.6	±	5.0
**Body composition**			
Age (yrs)	60.5	±	6.9
Height (cm)	155.7	±	4.5
Weight (kg)	59.1	±	7.1
BMI (kg/m2)	24.7	±	2.7
WHR	0.91	±	0.04
Heart rate (beat/min)	76.90	±	12.60
**Blood test**			
FBS (mg/dl)	126.4	±	18.3
PP2 (mg/dl)	206.7	±	54.3
HbA1c (%)	7.75	±	1.62
Total cholesterol	187.8	±	23.4
Triglyceride (mg/dl)	129.7	±	62.3
HDL-C (mg/dl)	51.0	±	12.5
LDL-C (mg/dl)	119.6	±	25.8

### Data analysis

To verify the hypothesis of this study, the data obtained from the experiments were computerized in accordance with the purpose of data analysis using SPSS for Windows. The means and standard deviations for all the variables were calculated. For the validity evaluation, correlation analysis was performed between the walking distance in the 6-MWT, heart rate during the recovery period (recovery heart rate) in the Tecumseh step test, and maximum oxygen uptake measured using the direct method. By using linear regression analysis of the measured items, the regression equation of the maximum oxygen uptake was obtained using the variables with the highest explanatory power. The significance level was set at *p* < 0.05.

## RESULTS

### Patients’ characteristics

A total of 54 participants were included, and their mean age was 60.5 ± 6.9 years (range, 50–70 years). The mean body mass index of the participants was 24.7 ± 2.7 kg/ m^2^, and the mean duration of diabetes mellitus was 6.6 ± 5.0 years (range, 0–20 years). The mean recovery heart rate was 76.9 ± 12.6 beats/min; 2-hour postprandial blood glucose level, 206.7 ± 54.3 mg/dL; glycated hemoglobin level, 7.75% ± 1.62%; TC level, 187.8 ± 23.4 mg/dL; TG level, 129.7 ± 62.3 mg/dL; HDL-C level, 51.0 ± 12.5 mg/dL; and LDL-C level, 119.6 ± 25.8 mg/dL.

### Cardiorespiratory fitness

The exercise capacities of the 54 patients with type 2 diabetes mellitus who underwent the exercise tests are shown in Table 2. The mean metabolic equivalence was 3.86 ± 1.17 metabolic equivalents (METs) at the ventilation threshold and 5.67 ± 1.32 METs at the maximum exercise load, and the maximum oxygen uptake was 19.1 ± 2.9 mL/(kg·min). At the ventilation threshold, the mean exercise load was 35.0 ± 15.5 W (maximum, 72.50 ± 17.3 W); mean heart rate, 101.9 ± 16.6 beats/min (maximum, 123.2 ± 17.2 beats/min); and systolic blood pressure, 137.1 ± 24.6 mmHg (maximum, 165.6 ± 21.5 mmHg). The average distance walked in the 6-MWT was 538.2 ± 54 m, and the recovery heart rate in the Tecumseh step test was 93.6 ± 11.6 beats/min.

**Fig.1.**
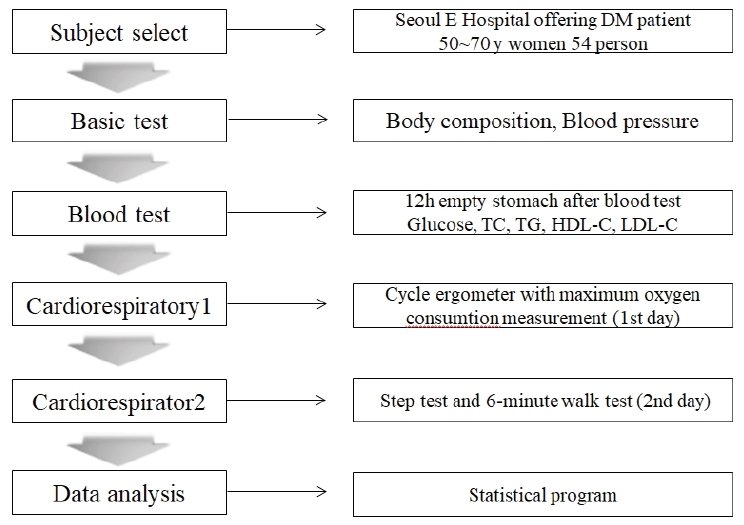
Flow chart of the experiment

**Table 2.** Physical capacity at the ventilatory threshold and maximal exercise. All data are presented as means ± standard deviations. MET, metabolic equivalent unit (1 MET=3.5 mL/kg/min), △Heart rate, ventilatory threshold heart rate – recovery heart rate; SBP, systolic blood pressure; DBP, diastolic blood pressure; 6-MWT, 6-minute walk test

Variable	Mean		S.D.
**Ventilatory Threshold**			
Load (watts)	35.0	±	15.5
METs	3.86	±	1.17
△Heart rate (beat/min)	101.9	±	16.6
SBP (mmHg)	137.1	±	24.6
DBP (mmHg)	79.3	±	10.1
**MAX**			
Load (watts)	72.5	±	17.3
${\dot{V}O}_{2max}$ (ml/kg/min)	19.1	±	2.9
METs	5.67	±	1.32
Heart rate (beat/min)	123.2	±	17.2
SBP (mmHg)	165.6	±	21.5
DBP (mmHg)	86.8	±	12.2
**6-MWT**			
Rest heart rate (beat/min)	75.1	±	11.3
End heart rate (beat/min)	108.7	±	15.0
Distance (m)	538.2	±	54.0
**Step Test**			
Rest heart rate (beat/min)	73.7	±	10.1
Recovery heart rate (beat/min)	93.6	±	11.6

### Correlation between ${\dot{V}O}_{2max}$ and 6-MWT distance and a regression equation for predicting ${\dot{V}O}_{2max}$ by the 6-MWT

**Fig.2.**
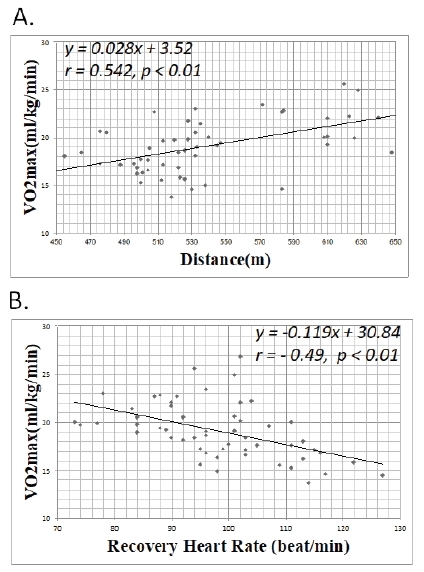
Correlation of the ${\dot{V}O}_{2max}$ with the 6-MWT distance and step test recovery heart rate. There was a significant correlation between the ${\dot{V}O}_{2max}$ and 6-MWT distance (Pearson’s correlation, *r*=0.54, *p*<0.01) and between the ${\dot{V}O}_{2max}$ and recovery heart rate (Pearson’s correlation, *r*=-0.49, *p*<0.01). Each point represents an individual patient. 6-MWT, 6-minute walk test

The correlation between the travel distance and the maximum oxygen uptake when other parameters were not taken into account is shown in Figure 2. The walking distance and maximum oxygen uptake of the subjects were highly correlated (Pearson correlation coefficient, *r* = 0.542). The final regression model obtained as a result of entering the independent variables using the step selection method is as follows, with significant coefficient values (*p* < 0.01).

***Y* (maximum oxygen uptake) = 14.986 + {0.025 × X1 (travel distance)} − {0.161 × X2 (weight)}**

### Correlation between ${\dot{V}O}_{2max}$ and step test heart rate and a regression equation for predicting ${\dot{V}O}_{2max}$ by the step test

The patients’ recovery heart rates and maximum oxygen uptakes were highly correlated (Pearson correlation coefficient, *r* = −0.49). The final regression model obtained as a result of entering the independent variables by the step selection method is as follows, with significant coefficient values (*p* < 0.01).

***Y* (maximum oxygen uptake) = 40.136 − {0.108 × X1 (recovery heart rate)} − {0.180 × X2 (weight)}**

## DISCUSSION

This study evaluated the cardiopulmonary capacity of patients with type 2 diabetes mellitus on the basis of cycle ergometer exercise test results, determined the maximum oxygen uptake equation by performing the 6-MWT and Tecumseh step test, and confirmed the validity and utility of the two tests using indirect measurements of the maximum oxygen uptake by comparing the results with those of the exercise load test.

Patients with diabetes mellitus are recommended to exercise properly for improving quality of life and promoting rehabilitation. However, in Korea, interest among such patients is minimal. In addition to not recommending active exercise, review of the therapeutic aspect is lacking and exercise programs for patients with diabetes mellitus have not been established yet. Although studies have been conducted with increasing interests in exercise regimens, only a few have estimated the maximum oxygen uptake in patients with type 2 diabetes mellitus^19,20^. This is because measurement of athletic ability by imposing a difficult exercise on the circulatory system is dangerous for patients with diabetes mellitus.

Considering the fact that patients with diabetes mellitus are at a high risk of complications of arteriosclerotic heart disease, the physical fitness of adult patients is likely to be lower than that of healthy adults. In fact, previous studies on this area have reported that the cardiopulmonary capacity of patients with diabetes mellitus is lower than that of healthy subjects^21,22^. Kunitomi et al.^23^ conducted an exercise test on female patients with diabetes mellitus without complications and found that the increase in heart rate or maximum oxygen uptake according to the exercise load was lower in the patients than in the healthy controls^23^. They reported that this is because of the deteriorating cardiopulmonary function due to coronary artery disease, which may be potentially present but not symptomatic, and the cardiovascular autonomic disturbance due to diabetes. In this study, the maximum oxygen uptake of the Korean patients with type 2 diabetes mellitus was 5.6 ± 1.3 METs. This indicates a level of fitness lower than the minimum intensity of 6 METs proposed by the American Sports Medicine Association.

Before encouraging diabetic patients to exercise, exercise prescription must first be given. Appropriate exercise prescription is performed by planning possible physical activities according to the individual’s ability systematically, but also according to the characteristics of the individual. Exercise prescription generally consists of FITT (frequency, intensity, type, and time). The most important and difficult problem in exercise prescription is determination of exercise intensity. If exercise intensity is too weak, exercise effect cannot be expected; if too strong, patients will not be able to exercise continuously. Therefore, to obtain the effects of exercise, exercise should be performed for a certain amount of time with appropriate exercise intensity. According to the American Society of Sports Medicine, the most appropriate intensity to improve cardiopulmonary function for healthy adults is 40–85% of the maximal exercise capacity. Methods of setting the exercise intensity include a heart rate-based method founded on linear proportion of heart rate and exercise intensity, methods based on fatigue awareness, a method based on maximum oxygen uptake^24^.

Among them, the measurement of maximum oxygen uptake is known to be the most accurate method for evaluating exercise capacity^25^. However, it is disadvantageous in that it is complicated and time-consuming to implement, the patient is put through physical difficulty during examination, and that accuracy is low when patients have low functional capacity. Low cardiopulmonary capacity not only increases the risk factors for diabetes treatment, but also lowers the ability to perform the work physically^26^. Posner et al.^27^ found that in direct measurement of maximum oxygen uptake in diabetic patients, it is difficult to obtain the actual maximum oxygen uptake due to fatigue of the large muscle groups, decreased lung function, and hypoglycemia^27^. In fact, in this study, when performing the exercise-load test for female diabetic patients ages 50–70 years, in one case, the exercise-load test could not be performed until the patient was exhausted, and even during the examination, careful observation was required, such as elevation of blood pressure or changes in the ECG.

Although the maximum exercise-load test is an accurate method for assessing cardiovascular endurance, due to problems such as expensive equipment and difficulty of the test itself, many studies have estimated the maximum oxygen uptake through maximal exercise without maximal load. The most widely used of these is Astrand-Ryhming, which estimates the maximum oxygen uptake on the basis of the heart rate obtained from the maximum bike exercise^25^. However, this method is not necessarily highly reproducible when applied to adults who are elderly or sick. In addition, studies in groups should include conditions such as economic efficiency, simplicity, stability, and feasibility. Therefore, studies have been conducted on possible methods to replace these exercises for pulmonary function test, and the 6-MWT or step test has been reported to be a potential replacement of the exercise-pulmonary function test.

Wyndlham reported a high correlation between the maximum oxygen uptake estimated through a 12-minute walk test and the directly measured maximum oxygen uptake (*r* = 0.87–0.94)^28^. Oja et al. found a correlation of *r* = 0.59–0.84 between the maximum oxygen uptake estimated on the basis of data obtained through a 2-km walk test and the maximum oxygen uptake obtained using a direct measurement method^29^. However, in general, when the equations for estimating the maximum oxygen uptake were applied to homogeneous groups or others, the range of correlation was too large (*r* = 0.19–0.90)^30-31^. On the other hand, in the present study, the maximum oxygen uptake measured through the 6-MWT had a correlation of *r* = 0.54, and *r* = 0.49, with a maximum oxygen uptake measured through the step test. This correlation is somewhat lower than those reported in previous studies that found that the correlation with maximum oxygen uptake estimated using the direct method was *r* = 0.59–0.84. This is thought to be due to the small number of subjects who participated in the study, and because the study was performed in relatively older patients.

In this study, regression analysis was performed for the assessment of the maximum oxygen uptake obtained using a cycle ergometer, and the validity of the 6-MWT and step test, all of which were statistically significant. In terms of indirectly assessing cardiopulmonary capacity, a regression analysis of the variables measured through the two tests showed that the step test had higher explanatory power of the maximum oxygen uptake than the 6-MWT. In future studies, more results are expected if the tests are conducted with a larger number of participants.

The mean travel distance of the 6-MWT was 538.28 ± 54 m, which was slightly lower than the 6-minute walking distance of 593 ± 57 m in the adult women reported by Alfredo et al. (2006). However, when considering the patients’ age and status in this study, they were thought to have walked fast, aligned with the intention of the test. Launkkanen et al.^6^ compared the walking speeds in a 2-km walk test and found that walking at 80%, as opposed to 50% and 60%, of the maximum heart rate most accurately estimated maximum oxygen uptake^11^. The degree of indirect estimation of the maximum oxygen uptake in diabetic patients between 50 and 70 years of age is generally lower than that in other age groups. The reason for this high estimation error is as follows: First, in the case of the elderly, the individual difference in whole-body endurance is large. Second, the individual differences in heart rate increase with age.

In Korea, the direct measurement method for evaluating the cardiopulmonary capacity of patients with type II diabetes is expensive, and requires advanced measurement techniques and a lot of time for measurement. In addition, exercise until exhaustion state under a high-intensity load requires strong motivation but is not suitable for persons without regular exercise habits, the elderly, or persons with illnesses. However, the 6-MWT and step test are simple, economical, and stable. For this reason, the 6-MWT has already been used as an exercise test in clinical practice, but the step test is not used. Research on the step test is mostly conducted in the general population, and little research has been conducted on the use of the Tecumseh step test. The Tecumseh step test is 20 cm in height with a lower exercise intensity than the normal step test, making it applicable to the elderly and sickly. In addition, it does not require advanced technology for measurement and could be used as a new method to measure the cardiorespiratory ability of patients in clinical practice.

As shown in the results of this study, the 6-MWT and step tests have statistically high reproducibility and validity. They are expected to be useful for assessing cardiopulmonary performance in patients with type II diabetes mellitus.
